# Fractal Analysis of Cervical Intraepithelial Neoplasia

**DOI:** 10.1371/journal.pone.0108457

**Published:** 2014-10-10

**Authors:** Markus Fabrizii, Farid Moinfar, Herbert F. Jelinek, Audrey Karperien, Helmut Ahammer

**Affiliations:** 1 Institute of Biophysics, Centre for Physiological Medicine, Medical University of Graz, Graz, Austria; 2 Unit of Breast and Gynaecologic Pathology, Institute of Pathology, Medical University of Graz, Graz, Austria; 3 Department of Pathology, Hospital of the Sisters of Charity, Linz, Austria; 4 Centre for Research in Complex Systems, Charles Sturt University, Albury, Australia; 5 School of Community Health, Charles Sturt University, Albury, Australia; State University of Maringá/Universidade Estadual de Maringá, Brazil

## Abstract

**Introduction:**

Cervical intraepithelial neoplasias (CIN) represent precursor lesions of cervical cancer. These neoplastic lesions are traditionally subdivided into three categories CIN 1, CIN 2, and CIN 3, using microscopical criteria. The relation between grades of cervical intraepithelial neoplasia (CIN) and its fractal dimension was investigated to establish a basis for an objective diagnosis using the method proposed.

**Methods:**

Classical evaluation of the tissue samples was performed by an experienced gynecologic pathologist. Tissue samples were scanned and saved as digital images using Aperio scanner and software. After image segmentation the box counting method as well as multifractal methods were applied to determine the relation between fractal dimension and grades of CIN. A total of 46 images were used to compare the pathologist's neoplasia grades with the predicted groups obtained by fractal methods.

**Results:**

Significant or highly significant differences between all grades of CIN could be found. The confusion matrix, comparing between pathologist's grading and predicted group by fractal methods showed a match of 87.1%. Multifractal spectra were able to differentiate between normal epithelium and low grade as well as high grade neoplasia.

**Conclusion:**

Fractal dimension can be considered to be an objective parameter to grade cervical intraepithelial neoplasia.

## Introduction

Prior to becoming invasive, cervical cancer is preceded by progressively worsening lesions that remain inside of the epithelium, known as cervical intraepithelial neoplasia (CIN). These precursor lesions are typically classified into: CIN 1 (“mild dysplasia”) or low grade, CIN 2 (“moderate dysplasia”), and CIN 3 (for a spectrum that includes “severe dysplasia and carcinoma in situ”) also referred to as high grade squamous intraepithelial lesions [1], [2].

For patients with CIN 1 lesions, the typical treatment is watchful waiting, because these lesions often turn back into normal tissue. In contrast, for patients with CIN 2 and 3 lesions, the recommended strategy is excision, intended to stop progress toward carcinoma, followed by intensified surveillance [3], [4]. Thus, cervical cancer is considered largely curable if caught before it progresses to invasive disease, and most national as well as international guidelines propose early detection, individualised treatment, prevention programs and follow-up procedures [3]–[6].

Currently, lesions are typically graded by pathologists using microscopes to assess tissue samples based on features such as the amount of dysplastic cells. CIN (synonyms: dysplasia, squamous intraepithelial lesion, SIL) is characterized by abnormal maturation and architectural abnormalities with atypical squamous cells showing nuclear atypia/pleomorphism. Based on the degree of architectural abnormalities and cytologic atypia, CIN is traditionally classified in low-grade (mild dysplasia), intermediate (moderate dysplasia) and high-grade (severe dysplasia/cancer in situ). While CIN 1 (mild dysplasia) shows low-grade architectural abnormality of atypical cells confined to the lower third epithelium, CIN 2 and CIN 3 are associated with markedly atypical squamous cells confined to more than half, but less than upper third of the epithelium (CIN 2) or even replace almost the entire native epithelium (CIN 3). Cervical intraepithelial neoplasia grade 2 (CIN 2) and grade 3 (CIN 3) are also regarded as high grade squamous intraepithelial lesions (HGSIL) according to the Bethesda- classification system [7]. In difficult and/or uncertain cases, immunohistochemical methods can be used to increase the accuracy of diagnosis [8].

In any case, grading depends on the expertise of the pathologist because these methods are semi-quantitative and not objective. Indeed there is no parameter in use that quantifies CIN grades based on an exact mathematical method [3]–[6].

One mathematical model that may be able to resolve this problem is fractal analysis. Fractal methods have been widely applied in medicine for many years and are especially relevant to the study of cancer due to the surface characteristics of the spreading tumor and tissue characteristics that may include protein deposits within the cytoplasm or staining anomalies with known stains or changes in nuclear morphology. Fractal dimensions have been used to assess tumor growth [11], [12], [13], chemotherapy-induced apoptosis [14], hematological cell phenotypes [15], grades of anal intraepithelial neoplasia [16], cerebral arteriovenous malformations [17], and oligodendrogliomas [18]. Recently, fractal analyses have been reviewed for chromatin in [19] and in neurosciences in [20], [21].

Some investigators have attempted to develop objective fractal analysis-based methods for grading CIN, but a practical and reliable method has not yet been found. Sedivy et al., for instance, concluded that the fractal dimension of single nuclei differed between CIN 1, CIN 2 and CIN 3 [9], but their method involves the very cumbersome task of extracting individual nuclei. Jayalalitha and Uthayakumar also used fractal methods to show how normal tissue could be divided from CIN lesions in general [10].

The current study proposes a new approach for using fractal methods to objectively grade CIN and normal tissue. Having a predefined ROI selected out of each tissue sample, we show how box counting can be applied to digital images of suspicious epithelium. In addition multifractal analysis was also investigated as differences in complexity throughout a biological image such as squamous epithelium can be attributed to multiple processes acting on the tissue during development and at different scales such that both microscopic and macroscopic influences determine the final structural attributes of the tissue. Fractal and multifractal analysis provide an easy, inexpensive and reliable method sensitive enough to establish an automated image diagnosis system from Haematoxylin-Eosin stained tissue samples.

## Methods

### Material Acquisition

**Figure 1:**
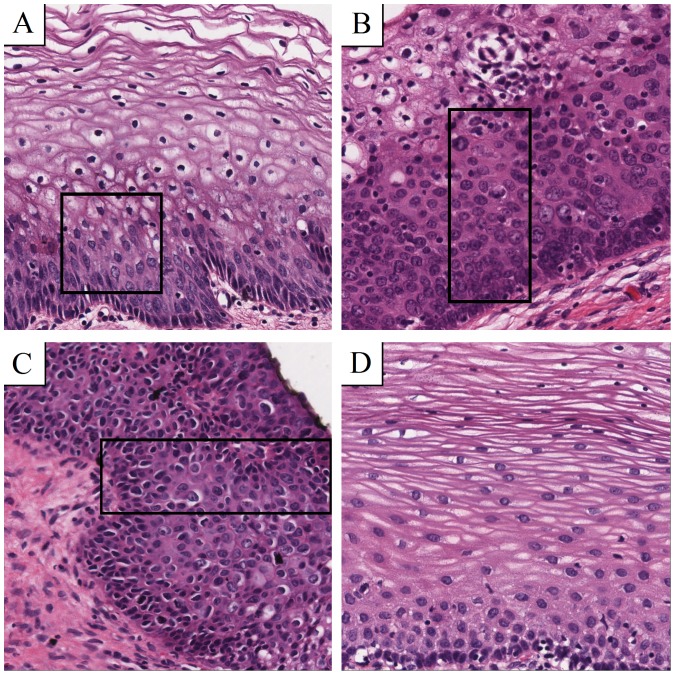
Morphology of CIN grades and normal epithelium. All samples are Haematoxylin-Eosin (H&E) stained and digitally scanned at a magnification of 40. Boxes show witch area to focus on. (A) Atypical cells in the first third are typically for CIN 1. (B) CIN 2 contains atypical cells in the lower two thirds of the epithelium. (C) If the whole epithelium is covered by atypical cells, the grade is called CIN 3. (D) Atypical cells are missing in normal epithelium.

Tissue samples, already described in a previous study were used for this analysis [22]. After Haematoxylin-Eosin (H&E) staining, a pathologist diagnosed the grades of CIN according to international guidelines. Regions of interest with constant size were chosen, focusing exactly on the suspicious part of the epithelium and avoiding background such as the object slide itself or non-epithelial tissue representing all grades of CIN as well as normal tissue to obtain best comparable and relevant image details for both ways of calculating fractal dimensions used later on. Overall, 46 samples were evaluated and categorized into 4 groups: CIN 1 (8 samples), CIN 2 (6 samples), CIN 3 (17 samples) and normal epithelium (15 samples). Figure 1 shows examples of these morphologies.

### Image acquisition and segmentation

Histological slices were digitally scanned using a digital whole slide scanner (Scan Scope T3, Aperio, Leica, Vienna) at a magnification of 40 (100000 pixel/inch). Images were saved in 24 Bit RGB colour format. To obtain comparable extracts out of the whole images, tiles of 1024×1024 pixels showing the suspicious epithelium were saved as TIFF files using ImageScope Viewer (Aperio) separately. Initially, the complete image of a histological slice was about 700 MB, tiles reduced to approximately 3.5 MB.

**Figure 2:**
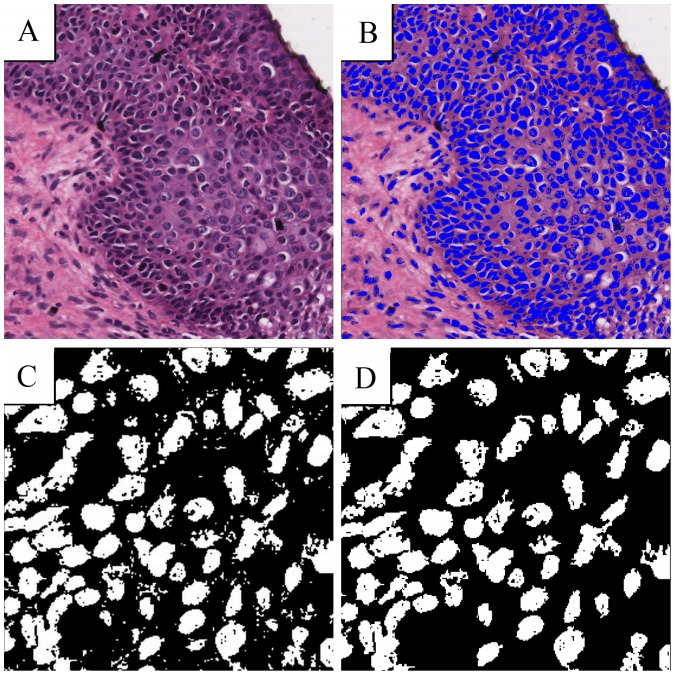
The use of RGB relative makes nuclei appearing blue (A). B shows the same image having set blue to white and all other pixels to black. The picture containing blobs (C) can be improved by erasing them to focus on the nuclei (D).

Segmentation and monofractal (global) fractal analysis was performed with the software IQM [25]. Multifractal analysis, which provides a multifractal spectrum and based on box counting was performed with public domain software Fraclac V2.5, and available as a Plugin to ImageJ [24], [26]. Nuclei can be seen very clearly using relative RGB segmentation with a ratio setting of 41 in IQM (Figure 2B). Binary images were created by setting the blue nuclei to white and every other part of the image to black. In order to improve image segmentation quality, small residual blobs were eliminated. Blobs were identified by the absence of adjacent pixels so that only nuclei remained for assessment [25]. Magnified sample images can be seen in Figures 2C and 2D.

### Box counting method

Theoretical fractals are self-similar objects following a scaling relationship or power law when certain measures are calculated at different scales. Biological objects may have fractal characteristics and are scale-invariant [23]. A measured value (e.g., length of an object) changes according to the resolution that the object is watched at. There exist several methods in order to estimate a value for the fractal dimension of objects in digital images, but the box counting method is inherently very well suited for that purpose. It uses boxes, which can be easily overlaid on an image's pixel grid. To determine the box counting dimension *d_BOX_*, the size *r* of the boxes building the grid is defined and the number of boxes *N(r)* covering the object in an image determined. The formal relation is:
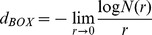



The limit cannot be calculated for digitized images, because of the discrete nature of the pixel grid. Therefore, an estimation of the fractal dimension is determined.

If the size of the box is exactly the length of an image's margin, *r* equals 1. In that case *N(r)* equals 1, too, because, certainly the object is inside this box. By halving the length (*r  = *1/2), four boxes result and the object could be overlaid by 1 up to 4 boxes, yielding *N(r)* to be 1 up to 4. The box length can further be divided into quarters (*r* = 1/4), eighths (*r* = 1/8), and so on, increasing the resolution step by step. If *N(r)* and *r* are double logarithmically plotted a scatter plot can be produced. Linear regression gives an approximation with a straight line and finally, the negative slope of this line equals *d_BOX_*.

### Multifractal method

We also used the basic box counting data gathering method to calculate the multifractal spectra. Multifractal spectra assess how scaling varies over an image. The parameter we used was the generalized dimension spectra, denoted *D_q_* vs *q*, calculated using FracLac for ImageJ [24], [26]. *D_q_* vs *q* spectra is calculated by setting an arbitrary range for *q,  = −10 to +10.75* with 0.25 increments. The multifractal spectrum has been most often estimated using the box counting method:
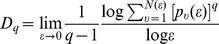



Here *ε* is the scaling factor, index *υ* labels the individual box and *p_υ_(ε)* denotes the relative weight of the *υ* ^th^ box. For finite data sets *p_υ_(ε)* is approximated by
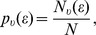
where *N_υ_(ε)* is the number of points falling into the *υ* ^th^ box [27].

### Statistics

Statistics were calculated using SPSS 20 (IBM, USA). In some cases it was possible to extract more than one area from the histological slice of one patient. To keep statistics valid, the median was calculated and counted as one sample. T-tests, ANOVA, Tukey HSD post hoc tests and Kolmogorov Smirnov-tests were used where applicable. Student's t-tests were performed for comparing low grade neoplasia (including CIN 1) to high grade neoplasia (including CIN 2 and CIN 3). In every case a *p*-value was considered to be significant if *p*<0.05 or highly significant if *p*<0.01. To evaluate sensitivity and specificity of the new method a confusion matrix assuming the pathologist's grading being the correct one was constructed.

## Results

**Figure 3:**
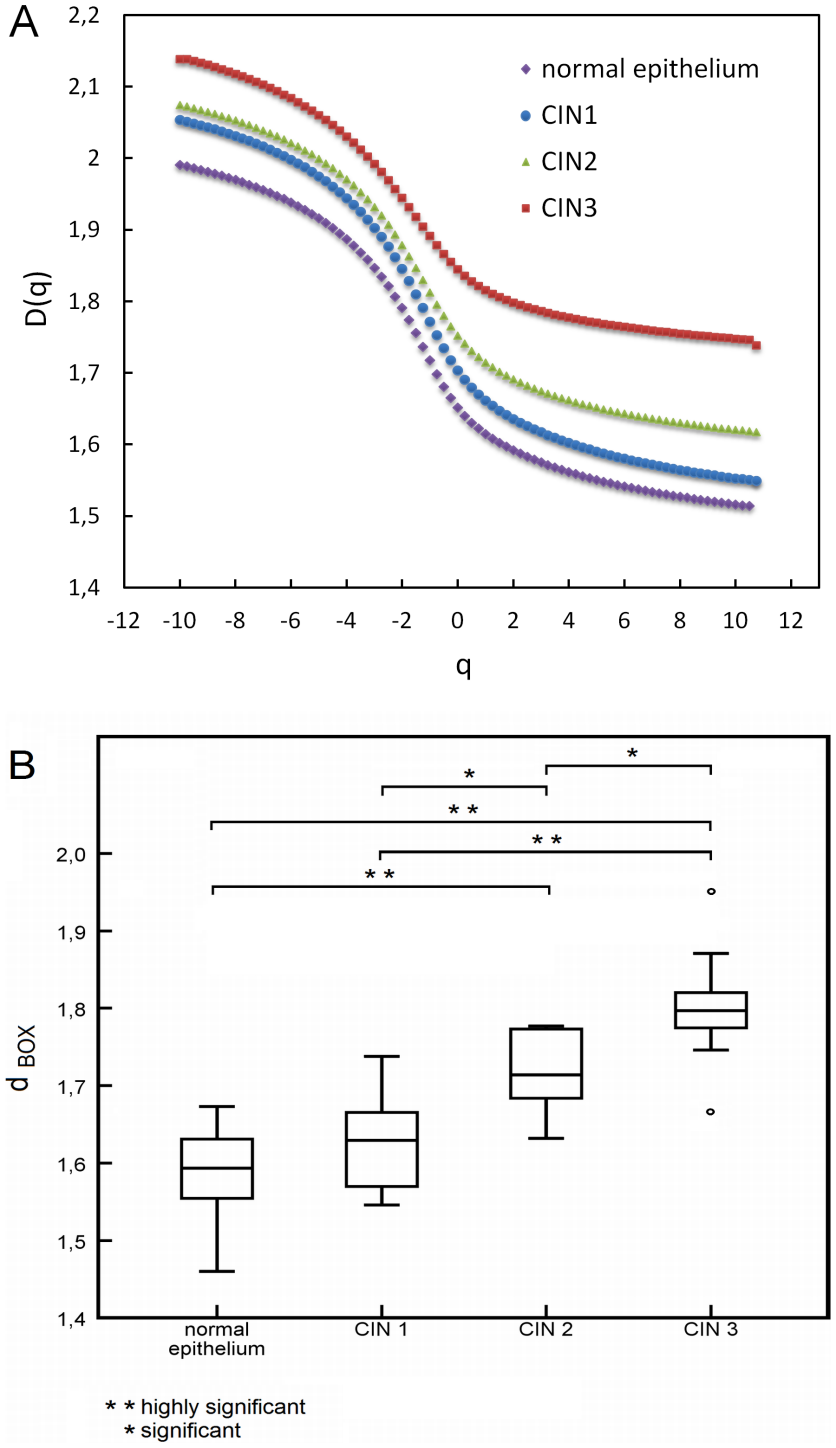
Relation between grades of neoplasia and normal epithelium. (A) Multifractal spectra and (B) Box plots of box counting dimension *d_BOX_*.

The *D_q_* vs *q* multifractal spectra we generated for the images indicated that there was some variation in the degree of multifractal scaling in between the neoplasia groups and between the neoplasia groups and the normal epithelium group. Based on ANOVA for the scales of *q* applied, there were significant differences between all groups and also between low and high grade neoplasia and normal epithelium. Tukey HSD post hoc tests indicated that for the group comparisons (CIN°1, CIN°2, CIN°3 and normal epithelium), CIN°3 was significantly different to normal epithelium (*p°*<°0.001), CIN°1 (*p°*<°0.01) and CIN°2 (*p°*<°0.05), and normal epithelium further significantly different to CIN°1 (*p°*<°0.05) and CIN°2 (*p°*<°0.01). These results held for all comparisons between -10°≤°*q°*≤°10.75, up to *q°>°*−7 when CIN°2 ceased to be significantly different to CIN°3, and *q°*>°−4.25 when no so significant difference could be seen between normal epithelium and CIN°1 (see Figure 3A). Based on the box counting dimension *d_BOX_*, however, a clear dependency emerged. Figure 3B shows box plots of the results. The lowest fractal dimension (*d_BOX_*° = °1.460) was found in normal epithelium and the highest (*d_BOX_*° = °1.9507) in CIN 3. Table 1 shows the minimum and maximum values as well as median values of *d_BOX_*, calculated out of *n* cases grouped in normal epithelium, low grade neoplasia, high grade neoplasia and CIN 1, CIN 2, CIN 3.

**Table 1:** Detailed values of fractal dimensions as well as the calculated values of minimum, maximum and median.

	normal epithelium	CIN 1	CIN 2	CIN 3	low grade neoplasia	high grade neoplasia
n	15	8	6	17	8	23
median	1,59	1,63	1,71	1,80	1,63	1,78
min	1,46	1,55	1,63	1,66	1,55	1,63
max	1,67	1,74	1,78	1,95	1,74	1,95
fractal dimension *d_BOX_*	*1,67*	*1,74*	*1,78*	*1,95*	*1,74*	*1,95*
	*1,67*	*1,67*	*1,77*	*1,87*	*1,67*	*1,87*
	*1,67*	*1,66*	*1,72*	*1,86*	*1,66*	*1,86*
	*1,65*	*1,64*	*1,70*	*1,83*	*1,64*	*1,83*
	*1,62*	*1,62*	*1,68*	*1,82*	*1,62*	*1,82*
	*1,61*	*1,58*	*1,63*	*1,82*	*1,58*	*1,82*
	*1,60*	*1,56*	*-*	*1,81*	*1,56*	*1,81*
	*1,59*	*1,55*	*-*	*1,80*	*1,55*	*1,80*
	*1,58*	*-*	*-*	*1,80*	*-*	*1,80*
	*1,57*	*-*	*-*	*1,79*	*-*	*1,79*
	*1,56*	*-*	*-*	*1,79*	*-*	*1,79*
	*1,55*	*-*	*-*	*1,78*	*-*	*1,78*
	*1,52*	*-*	*-*	*1,77*	*-*	*1,78*
	*1,51*	*-*	*-*	*1,77*	*-*	*1,77*
	*1,46*	*-*	*-*	*1,75*	*-*	*1,77*
	*-*	*-*	*-*	*1,75*	*-*	*1,77*
	*-*	*-*	*-*	*1,66*	*-*	*1,75*
	*-*	*-*	*-*	*-*	*-*	*1,75*
	*-*	*-*	*-*	*-*	*-*	*1,72*
	*-*	*-*	*-*	*-*	*-*	*1,70*
	*-*	*-*	*-*	*-*	*-*	*1,68*
	*-*	*-*	*-*	*-*	*-*	*1,66*
	*-*	*-*	*-*	*-*	*-*	*1,63*

Cases are grouped into normal epithelium and according to the grade of neoplasia.

The medians of *d_BOX_* increase according to the grade of neoplasia from 1.59 in normal epithelium to 1.80 in CIN 3 and 1.78 in high grade neoplasia.

Considering minimum and maximum values of *d_BOX,_* it can be seen that groups do overlap to some extent, e.g. the maximum of *d_BOX_* for normal epithelium reaches the median of *d_BOX_* for CIN 1.

All groups were normally distributed. ANOVA and Tukey HSD post hoc tests yielded significant results: CIN 1 was highly significantly different from CIN 3 (*p*<0.01), and normal epithelium was highly significantly different from both CIN 2 (*p*<0.01) and CIN 3 (*p*<0.01). In the significantly different category, CIN 1 differed from CIN 2 (*p* = 0.049) and CIN 2 from CIN 3 (*p* = 0.028).

The confusion matrix (Table 2) gives details about the reliability of the method. Comparing the CIN grade predicted by the box counting dimension to the pathologist's grading, corresponding results were achieved in 65.2% of the cases. CIN 3 matched in 82.4%, CIN 2 in 50%, CIN 1 in 50% and normal epithelium in 60% of cases.

**Table 2:** Confusion matrix comparing pathologist's results for CIN 1, CIN 2, CIN 3 and predicted group by box counting dimension *d_BOX_*.

			Group predicted				Total
			*normal epithelium*	*CIN 1*	*CIN 2*	*CIN 3*	
**Classification by the pathologist**	*n*	*normal epithelium*	9	4	2	0	*15*
		*CIN 1*	3	4	1	0	*8*
		*CIN 2*	0	1	3	2	*6*
		*CIN 3*	0	1	2	14	*17*
	*in %*	*normal epithelium*	60,00%	26,67%	13,33%	0,00%	100,00%
		*CIN 1*	37,50%	50,00%	12,50%	0,00%	100,00%
		*CIN 2*	0,00%	16,67%	50,00%	33,33%	100,00%
		*CIN 3*	0,00%	5,88%	11,76%	82,35%	100,00%

**Figure 4:**
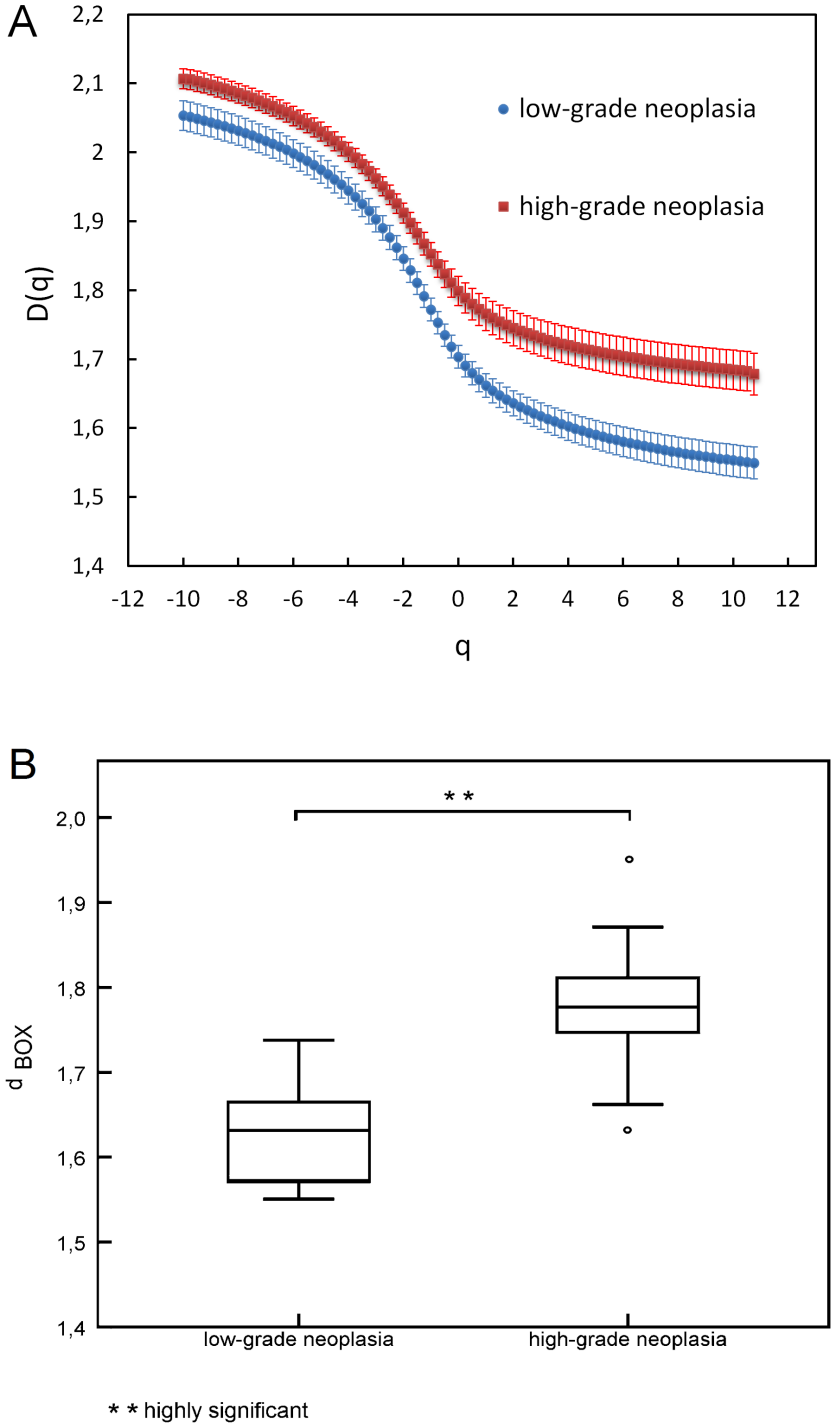
High grade and low grade neoplasia. (A) Multifractal spectra. Error bars represent standard errors of mean. (B) Box plots of box counting dimension *d_BOX._*

For further statistical examinations, two groups were formed: high-grade (CIN 2 and 3) and low-grade (CIN 1) neoplasia. Statistical comparison between low grade neoplasia and high grade neoplasia using multifractal spectra provided a highly significant result (Student's t-test, *p*°<°0.002) and is depicted in Figure 4A. For box counting dimension *d_BOX_*, Kolmogorov Smirnov-testing confirmed normal distributions and t°-°testing yielded a highly significant (*p*<0.01) difference between the two groups as can be seen in Figure 4B. Overall, the lowest value measured for the fractal dimension was 1.5460 (low-grade neoplasia) and the highest was 1.9507 in the group of high-grade neoplasia.

The confusion matrix of the high grade group and the low grade case yielded an overall success of 87.1% as can be seen in Table 3. Low-grade neoplasia matched in 87.5% of the cases and high-grade neoplasia matched in 86.96% of the cases.

**Table 3:** Confusion matrix comparing pathologist's results for low-grade and high grade neoplasia and predicted group by box counting dimension *d_BOX_*.

			Group predicted		Total
			*low-grade neoplasia*	*high-grade neoplasia*	
**Classification by the pathologist**	*n*	*low-grade neoplasia*	7	1	*8*
		*high-grade neoplasia*	3	20	*23*
	*in %*	*low-grade neoplasia*	87,50%	12,50%	100,00%
		*high-grade neoplasia*	13,04%	86,96%	100,00%

## Discussion

Recent studies by others have shown that quantitative CIN grading using fractal methods is possible, but those studies have had major limitations. One approach was to determine the fractal dimension of sub-images of single nuclei [9], but it is cumbersome and time consuming to segment and extract many nuclei from an image. In addition the form of the nuclei is dependent on the section and therefore slide processing is a confounding factor with this method. In another study the fractal dimension was used to discriminate between normal tissue and abnormal tissue (CIN 1, CIN 2, CIN 3) using the whole image of epithelium [10]. Unfortunately, the discrimination of the distinct grades of neoplasia was not shown in this study.

The methods we propose have an advantage over the single nuclei approach in being much easier to implement and incorporate information from the whole of the tissue. Image segmentation using a ROI to apply fractal analysis (common box-counting as well as multifractal spectra) also improves on the state of the art by presenting a high correspondence with the pathologist's grading despite a rather low number of images under investigation. It is a fact that there are actually only few cases, especially in the groups CIN 1 and CIN 2. However, the trend of increasing fractal dimension according to the grade of neoplasia is considerable and it can be assumed that further studies will confirm our findings. Multifractality is relevant to patterns in which a spectrum of fractal dimensions can be identified rather than a single global dimension. The theory and calculations behind multifractal measures are available elsewhere [24], [28]. Multifractal systems are common in nature and our analysis indicates that the type of lesions discussed in this paper also has these characteristics and may provide additional information to the clinician with reference to the scale of investigation. By using these methods we show, that it is possible to find significant or highly significant differences between all grades of CIN, between higher grades and normal epithelium and between low and high grade lesions as applied in clinical pathology classification. Although we found one potential limitation in that there was no statistically significant difference between normal epithelium and low grade neoplasia using the global box counting method. Multifractal analysis, however, did show significant differences between low grade neoplasia and normal epithelium and thus provides a more sensitive measure for certain scales. The sensitivity of differentiating between low grade neoplasia and normal epithelium images as well as low grade to high grade neoplasia using the multifractal spectrum highlights the differences that may be involved in the pathophysiological process that not only characterize the formation of the low grade neoplasia but suggests a different process that may be involved in the pathophysiological processes leading to high grade neoplasia. The limitation of the box counting method not to differentiate between low grade neoplasia and normal epithelium is not likely to cause problems when using automated image diagnosis in clinical practice, because surgery is inappropriate for low grade neoplasia as well as for normal epithelium. In addition screening is carried out annually and therefore the likelihood of detecting the low grade neoplasia increases. This is further improved by low grade neoplasia being significantly different to the high grade neoplasia shown in our experiments. As with all clinical studies of this nature, the pathologist diagnosis may in some instances be inaccurate, especially when identifying differences between normal and grade CIN 1. As such the classification system used by the pathologist may also have some bearing on the accuracy of any fractal analysis in the classification task.

## Conclusions

We have demonstrated that objective parameters for grading of CIN can be found by applying the proposed methods including image segmentation and fractal analyses. Compared to other approaches, the proposed methods are fast, easy and reliable. The next step in developing this technology is to establish a clinical standard based on fractal measures. Involvement of a larger number of pathologists as well as a larger number of images could help to reinforce this effort. Since the method seems to be very appropriate, a comparison study of the proposed objective method and the rather subjective grading in clinical practice could be performed. Another application could be the development of software that yields a specific prognosis, e.g. using fractal dimensions to predict if a CIN 1 lesion is more likely to turn into CIN 2 or might become normal tissue again, or to further characterize changes within the low grade neoplasia and specifically CIN 3 identified in multifractal spectra.
